# Constructing Topic Models of Internet of Things for Information Processing

**DOI:** 10.1155/2014/675234

**Published:** 2014-07-09

**Authors:** Jie Xin, Zhiming Cui, Shukui Zhang, Tianxu He, Chunhua Li, Haojing Huang

**Affiliations:** ^1^The Institute of Intelligent Information Processing and Application, Soochow University, Suzhou 215006, China; ^2^Provincial Key Laboratory for Computer Information Processing Technology, Soochow University, Suzhou 215006, China; ^3^State Key Laboratory for Novel Software Technology, Nanjing University, Nanjing 210093, China

## Abstract

*Internet of Things* (IoT) is regarded as a remarkable development of the modern information technology. There is abundant digital products data on the IoT, linking with multiple types of objects/entities. Those associated entities carry rich information and usually in the form of query records. Therefore, constructing high quality topic hierarchies that can capture the term distribution of each product record enables us to better understand users' search intent and benefits tasks such as taxonomy construction, recommendation systems, and other communications solutions for the future IoT. In this paper, we propose a novel *record entity topic model* (RETM) for IoT environment that is associated with a set of entities and records and a Gibbs sampling-based algorithm is proposed to learn the model. We conduct extensive experiments on real-world datasets and compare our approach with existing methods to demonstrate the advantage of our approach.

## 1. Introduction 

The* Internet of Things* (IoT) is a novel paradigm that is rapidly developing in the scenario of modern wireless telecommunications and the information age. The ubiquitous of IoT technology especially in embedded devices has led to smart systems that affect people's daily life. The concept behind IoT is that a variety of objects around us can interact and work with each other to pursue common goals [1].

There are several popular services among IoT such as online shopping or goods recommendation systems. An ideal e-commerce website should correctly understand user's query intent and return satisfactory searching results as fast as possible; a favorite recommendation system should exactly predict user's individual preference, meaning the application must be cognizant of the topics behind every piece of product. The fact is tens of thousands of digital products/records within IoT constantly update and new records are generating every day. How to manage those data, how to define right records categories, those two questions will bring fresh challenges to the IoT industry.

A variety of existing works are dedicated to constructing topic or concept hierarchies from text data in the Internet. One innovative approach is to learn a topic model based on probability statistics. Traditional topic models treat each document as a bag of words and predict the topic through the word distributions. It has been widely used in fields such as taxonomy construction [2], concept extraction [3], citation analysis [4], and social network regularization [5]. LDA [6] and PLSA [7] are the most well-known ones and several different topic models are proposed as extensions. For example, Link-LDA [4] and HTM [8] are designed to deal with documents with hyperlinks, citations, and other forms of link information; author model (AM) [9] and author topic model (ATM) [10] deal with author related academic paper search; Link-PLSA-LDA [11] and MB-LDA [12] are designed to model documents like blogs or twitters.

Although these models perform well in certain domains, they are not appropriate in our case for two reasons. First, most of the records consist of short text like tweets, so it is sometimes difficult to discover the topic through only a few tokens. But unlike tweets and other short texts, these records exist behind the query interface of certain websites and can only be obtained through keywords querying. Records from the same keyword query have some connections between each other and together they represent common topics. In other words, the word distribution of a single record can be regarded as a mixture latent topic distribution of current record and its “neighbor” records (records extracted from the same keyword queries), and the overall query records list might finally determine the topic of one record. Second, one product record usually is associated with plenty of attributes that describe the detailed features. For example, a book record has attributes like author and publisher; a movie record contains attributes like casts and director; a research paper record is associated with authors and publication. The truth is that almost any record is associated with some set of real-world entities, which is the foundation of IoT. Despite the fact that previous works have involved one or two types of entity relations in their topic models, like author relationship in ATM, they can only deal with certain domain documents. While our records associate with multiple objects, our topic model should be more general to conduct the topic analysis in diverse domains.

To further explain our goal, we need to know the term distributions for each entity or topic-entity pair. Specifically, let *z*, *e*, *w* denote a topic, an entity, and a word, respectively; our goal is to design a topic model that can solve the problem like *P*(*w*∣*z*), *P*(*w*∣*e*), and *P*(*w*∣*e*, *z*).

The dependencies among topic, entity, and word could help the system to gain a better understanding of the model. For example, in a collection of computer science research papers, we could know the basic concept of data mining through the word distribution of  *P*(*w*∣Data  Mining); we could know a specific author A's research interest in data mining area by analyzing the word distribution of  *P*(*w*∣A, Data  Mining); we can further compare his data mining related work with other researchers like B in the same field by comparing *P*(*w*∣A, Data  Mining) with *P*(*w*∣B, Data  Mining).

In addition, topic model that involves entity distributions can be more accurate since it might avoid some mistakes caused by traditional models. In most previous works, they do not care for the distribution of *P*(*w*∣*e*, *z*) but directly assume that either *P*(*w*∣*e*, *z*) = *P*(*w*∣*e*) or *P*(*w*∣*e*, *z*) = *P*(*w*∣*z*) by introducing different types of conditional dependency relations among topics, entities, and words. For instance, both LDA and Link-LDA believe that *P*(*w*∣*e*, *z*) equals to *P*(*w*∣*z*); ATM, on the other hand, assumes that *P*(*w*∣*e*, *z*) = *P*(*w*∣*e*). However, such “seems reasonable” assumptions may not always be correct in many cases. Recall the previous example, if *P*(*w*∣*e*, *z*) = *P*(*w*∣*e*)  holds, we can obtain that *P*(*w*∣A, Data  Mining) = *P*(*w*∣A, Machine  Learning), but this equation does not hold since authors seldom use the same terms in papers from different topics. Meanwhile, if *P*(*w*∣*e*, *z*) = *P*(*w*∣*z*)  holds, means *P*(*w*∣A, Data  Mining)  is equal to *P*(*w*∣B, Data  Mining), but obviously different authors use different terms due to writing habits even doing the same research topic. To this point, it is really necessary for us to model *P*(*w*∣*e*, *z*) directly to find the correlation of words between a pair of an entity and a topic.

To trickle the above challenges, we propose a novel topic model to analyze the topics of querying records in IoT environment named* record entity topic model* (RETM). This model is a promotion of LDA model and argues that each record can be identified as a word distribution over a set of entity-topic pairs. Furthermore, it points that “neighbor” records should have some impact on the topic distribution of one record, since they come from the same query list so that they have great topical similarities. In this case, these improvements make the model more flexible and specific.

The main contributions of our work are as follows. (1) We studied an important practical problem of modeling the topics for IoT industry using the latent relationships among querying records. To the best of our knowledge, this is the first work to introduce such issue. (2) We improve our model by adding entity relative dependency and emphasize the correlation of entity-term and topic-term distributions. We also adopt Gibbs-sampling-based algorithm to learn the model. (3) We conduct complex experiments on real-world dataset and compare with some state-of-the-art topic models. Results show that our method performs well in certain aspect and has some advantages.

The rest of this paper is organized as follows. We begin by reviewing the related works in the next section. After that, we introduce the overall model by giving the problem statements and describing the generation process as well as how to inference and learning the model in Section 3. The experimental results are addressed in Section 4 and conclusions are drawn in Section 5.

## 2. Related Work

Latent topic modeling has become very prevalent as a completely unsupervised technique for topic discovery. PLSA (probabilistic latent semantic analysis) [7] and LDA (latent Dirichlet allocation) [6] are two most well-known models in large document collections. The key idea of them is to represent topics as distribution of words and the document is a mixture over hidden topics so that it can be processed in this lower-dimensional space. However, the disadvantage of the two models is that they treat each document as an independent one and ignore the obverse linking relationships, so a lot of researches have been done while making use of multiple typed links in different ways.

The original purpose of topic models is to process plain text of documents. Recently, more attention has been paid in respect of topic discovery with hypertexts or citation information. Link-LDA [4] is designed specifically for scientific publication with citations, which regards one document as a combination of a bag of words and a bag of citations. It provides an efficient model to understand the distribution of links. Dietz et al. [13] propose a topic model for citation analysis, in which the content of a citing document is assumed to be generated by mixing the topic distributions of the cited documents. It is the first time to study the relations of citing paper and cited paper to a published paper. Link-PLSA-LDA [11] combines PLSA and LDA together into a single framework, which produces a bipartite graph to model the connections of citing documents to cited ones. Another model called HTM [8] argues that the content of a document is determined by its own topic distributions as well as by its cited documents', which is fairly similar to our model where we seek the background topics from the “neighbor” records. However, all the above models are particular designed for scientific publications, which are extremely different from querying records in structure and hardly applied to other domains.

In addition to review the hyperlinks between the documents, some researchers proposed models that concentrate on the relations of author. For instance, Mccallum [9] intends to model the author interests with a one-to-one correspondence between topic and authors in multilabeled documents, so each label could be represented as an entity. The ATM [10] integrates the authorship into the topic model, which addresses the task of modeling corpora annotated with the ids of multiple people who authored the documents. ArnetMiner [14] extends the aforementioned work by adding conference relations to its model. It tries to mine the academic social networks simultaneously from the aspects of papers, authors, and publication venues. Still all these models are limited to academic publications like mentioned above; in addition, they all treat *P*(*w*∣*e*, *z*) = *P*(*w*∣*z*), which is usually not the case.

Some works pay attention to model short text documents like records in our case. For example, PatentMiner [15] is proposed to mine the topic of heterogeneous patent network involving several types of objects like companies, inventors, and technical contents. However, it is uniquely designed for patent topic discovery and cannot be used for more general records of IoT, and in addition, all the patent records are gathered from databases, which is quite different from querying records in our case. Wang et al. [16] successfully discover the topics from web tables by summarizing the content of each row with columns. His dominant theme is similar to our model since we also argue there is a background topic that impacts the distribution of single record topics. However, instead of using probabilistic topic model, his work adopts a Hearst pattern-based method. MB-LDA [12] is designed for topic mining of microblogs or tweets, which takes both contact relation and document relation into consideration. Nevertheless, the structure and relations of tweets are significantly different from the records we are talking about. One model that particular points out the importance of differentiating term distribution from entity distribution in a topic model is ETM [17], who can explicitly model  *P*(*w*∣*e*, *z*). However, besides the previous explanations we made to illustrate the particular characteristics of our case, ETM has paid great attentions on determining the shared asymmetric Dirichlet priors. Conversely, our method tries to balance between entity distributions with topic distribution obtained from local topic or overall topics, so we decide to adopt symmetric Dirichlet priors, which is enough in our case. In addition, the two models have entirely different generative process.

## 3. Record Entity Topic Model

In this section, we firstly give the definition of RETM and then explain the generation process and finally provide a Gibbs-sampling-based learning algorithm to infer the model.

### 3.1. Overview of the Problem

Records we mentioned are obtained from keywords querying over the Internet. They are dynamic, highly qualified, and exist in the hidden databases. Each record consists of several attributes that describe all the associated features of one unique object. Most records involve multiple entities in the attributes, such as the attributes* director* and* production company* in Table 1. A collection of entity related records is the basic input of RETM.

**Definition 1 .**

Each record *r* is associated with a term vector, where each *w*_*d*,*i*_ is selected from a vocabulary database *W* and an entity vector  *E*_*d*_, which is chosen from a set of entity of size *E*. Let a collection of *R* records (*r* ∈ *R*) is determined by *R* = {〈*w*_1_, *E*_1_〉, 〈*w*_2_, *E*_2_〉,…, 〈*w*_*R*_, *E*_*R*_〉}.

The goal of this paper is to identify word patterns for each pair of an entity and a topic, that is, to find the precise word distribution over topic *z* and entity *e* to get *P*(*w*∣*e*, *z*), which also satisfies a multinomial distribution with parameter *φ*_*e*,*z*_. The notation of the whole paper is present in Notation section. Note that the entity we used in our model is gained from some well-known corpus such as DBpedia [18] or Probase [19]. Unlike ETM or other topic models, we assume that all the entities are following a multinomial distribution *δ*_*d*_, which are not generated but can be easily calculated with the help of modern knowledge base.

### 3.2. Generative Process of RETM

**Figure 1:**
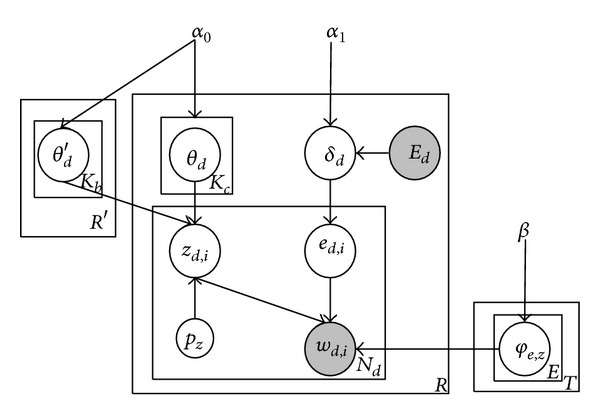
A graphical representation of RETM.

Figure 1 reveals the graphic representation of RETM. The core idea of this model is that firstly, different entities can be represented by different word distributions, and the words used to describe an entity can be changed with the topic. For example, Guo Jingming is known as a famous writer in the *Book* domain, but he is often identified as the director of movie* Tiny Time* in the* Movie* domain. In other words,  *P*(*w*∣*e*_*m*_, *z*) ≠ *P*(*w*∣*e*_*n*_, *z*) if *e*_*m*_ ≠ *e*_*n*_  and *P*(*w*∣*e*, *z*_*m*_) ≠ *P*(*w*∣*e*, *z*_*n*_) if  *z*_*m*_ ≠ *z*_*n*_. Secondly, the topics of one record could be determined based on the current record itself or together with its “neighbor” records. For example, in Table 1, judging from one record, we might say that it is a science fiction movie, but combined with the remaining two records from the same query, we can say that all these records are American movies of extraterrestrial for sure. That is the reason why we particularly studied the overall background topic in RETM.

Algorithm 1 explains the generative process of RETM. Note that each record has its own topic, and we name them* local topics*; one record and its “neighbor” records from the identical query list together produce some topics and we name them* background topics*. They all originate in the same vocabulary source and there are *K*_*c*_ local topics and *K*_*b*_ background topics, respectively. Therefore, for each record *d*, multinomial distributions *θ*_*d*_  and *θ*_*d*_′ over topics can be drawn from a Dirichlet prior with *α*_0_. Suppose that the probability of choosing local topics is *p*_*z*_ and the probability of choosing background topics has to be 1 − *p*_*z*_. By observations, we find that once the value of attributes consists of short tokens, record *d*'s topic mostly depends on the distribution of local topics. On the contrary, if the record contains long texts such as abstract information of a paper or the reviews of a product, *d* largely relies on the background topics. For convenience, we define a flag variable *t* ∈ {0,1}, where *t* = 1 means the multinomial distribution *θ*_*d*_ of a word  *z*_*i*_ is determined by local topics whereas *t* = 0 means *θ*_*d*_′  is selected. At the same time, multinomial distribution *δ*_*d*_ over entity set *E*_*d*_ can be drawn from a Dirichlet prior with  *α*_1_. Unlike other topic models which usually select an entity directly from the entity sets, we construct our model that depends on a record-specific multinomial distribution  *δ*_*d*_. This stems from the fact that each entity tends to have different weights in generating a record. For example, casts and the director dedicate differently to a movie record; if a research paper has multiple authors, the lead author possibly makes more effort than the others. Next, to generate each word in records, a topic *z*_*d*,*i*_ is drawn from the determined *θ*_*d*_ or  *θ*_*d*_′, and an entity *e*_*d*,*i*_ is drawn from *δ*_*d*_. After that, a word *w*_*d*,*i*_ can be generated through Gibbs sampling based on an entity and topic specific multinomial distribution *φ*_*e*_*d*,*i*_,*z*_*d*,*i*__ that has a Dirichlet prior *β*. In a word, each term is related to a pair of entity and topic and the generative process is the result of their joint actions. Here, the values of *w*_*d*,*i*_  and *E*_*d*_ can be derived from observations and parameters like *α*_0_, *α*_1_, and *β* can also be obtained from empirical statistics. Others parameters like *θ*_*d*_, *δ*_*d*_, *z*_*d*,*i*_, *e*_*d*,*i*_ and *φ*_*e*_*d*,*i*_,*z*_*d*,*i*__  are all unknown latent variables that needs to infer.

### 3.3. Model Learning and Inference

Now we can infer the topic of querying records through probability statistics based on previously known data and the generative process. Researchers usually employ approximate techniques to estimate the LDA parameters such as Variation Expectation Maximization [20], Expectation Propagation [21], or Gibbs sampling [22]. In this work, we intend to use a Gibbs-sampling-based method for the posterior distribution of the latent variables.

**Figure 2:**
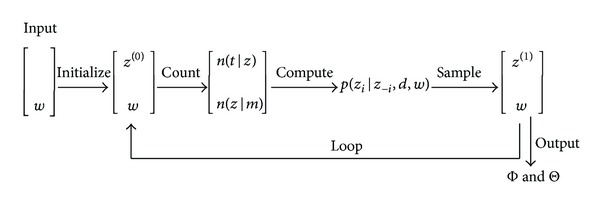
A brief illustration of Gibbs sampling process.

Gibbs sampling is a Markov chain Monte Carlo (MCMC) algorithm for obtaining a sequence of observations which are approximated from a specified multivariate probability distribution. It generates a Markov chain of samples, each of which is correlated with adjacent samples. The output parameters can be estimated iteratively until convergence.  Figure 2 gives a brief illustration of this entire process.

Depending upon this process, we could repeatedly sample the entity-topic pair for each word, in case of the assignment of all the rest words (*Z*, *ε*) along with the priors parameters. At this point, the conditional posterior of assignment (*e*_*d*,*i*_, *z*_*d*,*i*_) to the *i*th word *w*_*d*,*i*_  in record *d* is

(1) P(zd,i,ed,i,t ∣ wd,i,Z∖d,i,ε,Φ,pz)  ∝P(wd,i ∣ zd,i,ed,i,Z∖d,i,ε,Φ)  P(zd,i ∣ Z∖d,i,Φ)P(t ∣ pz)  P(ed,i ∣ ε∖d,i,Φ),

where subscript “∖*d*” denotes a quantity excluding data from position *i* in record *d* in order to eliminate the influence of current position word tokens.

Formula (1) is comprised of three terms that we can calculate separately. Firstly, term 3 denotes the probabilistic distribution of one entity in record *d* given all its associated entity sets except *i*th token in record *d*. For records set with *R* records, the prior over *δ* = (*δ*_1_,…, *δ*_*R*_)  is also assumed to be a symmetric Dirichlet with concentration parameter *α*_1_. Thus, given corresponding entity assignments *ε* = {*e*_*d*_}_*d*=1_^*R*^ in |*E*_*d*_|  entities vocabulary set, the conditional posterior of one main topic *k* of certain record *d* can therefore be written in the form

(2) P(ed,Nd+1=k ∣ ε,α1)=∫dδdP(k ∣ δd)P(δd ∣ ε,α1)=Nk|d+α1/|Ed|Nd+α1,

where *N*_*k*∣*d*_ = *n*_*d*_  denotes frequency of occurrence of topic *k* to all topics associated with record *d* and  *N*_*d*_ = ∑_*k*_ *N*_*k*∣*d*_. Note that each record can contain a few duplicated topics, but the main topics cannot be repeated.

It is clear that the probability of excluding entity *e*_*d*,*i*_  at current position is equal to total probability divided by the probability of excluding the word at *i*th position and  *e*_*d*,*i*_ = *k*, which we represent as *p*_−*di*_, where

(3) $$\begin{array}{c} p_{-di}=\overset{R}{\underset{d=1,d\neq di}{\prod }}\frac{\Delta \left(n_{d,-i}+\alpha_{1}\right)}{\Delta \left(\alpha_{1}\right)}, \end{array}$$

and the distribution probability *p*_*i*_ of *e*_*d*,*i*_  is

(4) $$\begin{array}{c} p_{i}=\frac{\Delta \left(n_{di}+\alpha_{1}\right)}{\Delta \left(\alpha_{1}\right)}, \end{array}$$

so that

(5) P(ed,i ∣ ε∖d,i,Φ)=P(ed,i ∣ εd,i,Φ)Δ(ndi,−i+α1)/Δ(α1)∝Ned,i ∣ d∖d,i+(α1/|Ed|)Nd−1+α1,

where *N*_*e*_*d*,*i*_∣*d*_^∖*d*,*i*^  is the number of word tokens assigned with entity *e*_*d*,*i*_  except *i*th token in record *d*. In addition, as defined in Dirichlet distribution, there is

(6) $$\begin{array}{c} \frac{1}{\Delta \left(\alpha \right)}=\frac{\Gamma \left(\sum_{v=1}^{V}\alpha_{v}\right)}{\prod_{v=1}^{V}\Gamma \left(\alpha_{v}\right)}, \end{array}$$

where *V* stands for the size of vocabulary set and meanwhile, we have *n*_*di*_ = *n*_*di*,−*i*_ + 1 according to our definition. At this point, we can infer (5).

For the second term in (1), as mentioned previously, the main topic may come from local topic or overall background topics. In the case of the Bernoulli distribution, the probability distribution over *t* can be written in the form

(7) $$\begin{array}{c} P\left(t\;|\;p_{z}\right)={\left(p_{z}\right)}^{n_{t}=1}{\left(1-p_{z}\right)}^{n_{t}=0}, \end{array}$$

where *t* is generated from Bernoulli (*p*_*z*_). Since word *w*_*i*_ is generated from local topic *z*_*d*,*i*_  if *t* = 1, according to Figure 1, there are *K*_*c*_ topics *Z* = {*z*_1_, *z*_2_,…, *z*_*k*_*c*__},  we have

(8) $$\begin{array}{c} P\left(z_{d,i}\;|\;Z_{\setminus d,i},\Phi \right)P\left(t\;|\;p_{z}\right)\propto \frac{N_{z_{d,i}\;|\;d}^{\setminus d,i}+\left(\alpha_{0}/K_{c}\right)}{N_{d}-1+\alpha_{0}}p_{z}. \end{array}$$

And if *t* = 0, word *w*_*i*_ is generated from background topic  *z*_*d*,*i*_′. Since there are *K*_*b*_ topics *Z*′ = {*z*_1_′, *z*_2_′,…, *z*_*k*_*b*__′}, we obtain

(9) $$\begin{array}{c} P\left(z_{d,i}\;|\;Z_{\setminus d,i},\Phi \right)P\left(t\;|\;p_{z}\right)\propto \frac{N_{z_{d,i}^{\prime }\;|\;d}^{\setminus d,i}+\left(\alpha_{0}/K_{b}\right)}{N_{d}^{\prime }-1+\alpha_{0}}\left(1-p_{z}\right), \end{array}$$

where *N*_*z*_*d*,*i*_∣*d*_^∖*d*,*i*^ or *N*_*z*_*d*,*i*_′∣*d*_^∖*d*,*i*^ is the number of word tokens assigned with topic *z*_*d*,*i*_or  *z*_*d*,*i*_′  except *i*th token in record *d*. Meanwhile, we take the same methods for determining *P*(*z*_*d*,*i*_∣*Z*_∖*d*,*i*_, Φ) as term 3.

We now determine the first term in the objective formula. Note that the distribution of words in every record is regarded as a mixture of topic-entity pair distributions. Given *K* topic entity pairs whose distribution *φ*_*e*,*z*_ is a multinomial distribution with a Dirichlet prior *β*, the word distribution probability that not include the word at position *i* and associated pair <*z*_*i*_, *e*_*i*_ > = *k* is in the form of

(10) $$\begin{array}{c} P\left(w_{i}\;|\;Z_{\setminus i},\varepsilon_{\setminus i},\Phi \right)=\overset{K}{\underset{k=1,\;\;k\neq \langle z_{i},e_{i}\rangle }{\prod }}\frac{\Delta \left(n_{k,-i}+\beta \right)}{\Delta \left(\beta \right)}, \end{array}$$

where *n*_*k*,−*i*_ stands for the number of tokens assigned with the pair 〈*z*_*i*_, *e*_*i*_〉 other than the word *w*_*i*_. Next, the probability of 〈*z*_*i*_, *e*_*i*_〉 = *k* can be written in the form of

(11) $$\begin{array}{c} P\left(w_{i}\;|\;z_{i},e_{i},\Phi \right)=\frac{\Delta \left(n_{k}+\beta \right)}{\Delta \left(\beta \right)}, \end{array}$$

where *n*_*k*_ is the number of word tokens that are assigned to the *k*th entity topic pairs.

Finally, the expression of first term in (1) takes the form

(12) P(wi ∣ zi,ei,Z∖i,ε∖i,Φ) =∏k=1,k≠〈zi,ei〉KΔ(nk,−i+β)Δ(β)·Δ(nk+β)Δ(β) =∏k=1KΔ(nk,−i+β)Δ(β)·Δ(β)Δ(n〈z,e〉=k,−i+β)·Δ(nk+β)Δ(β) ∝Δ(nk+β)Δ(n〈z,e〉=k,−i+β) =Γ(∑v=1V(nk,−iv+βv))∏v=1VΓ(nk,−iv+βv)·Γ(∑v=1V(nkv+βv))∏v=1VΓ(nkv+βv) =nk, −iv+βv∑v=1V(nk,−iv+βv),

given that

(13) $$\begin{array}{c} \Gamma \left(a+1\right)=a\Gamma \left(a\right),\\ n_{k}^{v}=n_{k,\;\;-i}^{v}+1. \end{array}$$

So far, we have obtained three terms in (1), which are the objective of this paper. With the known observed variables, once we obtain the entity topic pair assignments of each word, we can derive the distribution of word tokens over this pair, namely,  *φ*_*e*,*z*_. Then we can estimate the parameters like *θ*_*d*_, *θ*_*d*_′, and *δ*_*d*_  accordingly to get the distribution of each record over entity topic pairs to ultimately find the most possible topic for each record through analysis.

## 4. Experiments

### 4.1. Datasets

We make use of two large-scale datasets for the evaluation of our model, both of which are extracted from real-world data sources. One dataset is named* BOOK*, and all the records are crawled from Soochow University library bibliographic query system (http://lib.suda.edu.cn) through LAN. We pick up 2000 nonrepeating records of computer science books manually and each record has attributes that include* book name*,* author name*,* ISBN*,* press*,* price,* and* overview*. Owing to the demand of constructing background topics, we keep the URI information about records in the same query lists in the form of RDF. Another dataset is from ACM research paper data source, namely,* PAPER*. It contains 38469 published papers about information system retrieved in 2002–2011 years in 2012 September according to the classic category (download from: http://www.datatang.com/datares/go.aspx?dataid=619787). Each metadata consists of the corresponding* citation network*,* keywords*,* title*,* date of publication*,* journals, and abstract*. In order to facilitate the evaluation, we pick up the top 19235 records in our experiment, which is perfect to test our model, since it contains the associated keywords data. However, it does not contain the information about querying so it is not suitable for testing the background topic part.

To testify the impact of entity distribution on the main model, we use DBpedia Soptligh [23] to preprocess the data from two datasets. DBpedia Soptligh is a high-performance online entity extraction and disambiguation service that links all the extracted entity to Wikipedia, and in addition, with the help of DBpedia, we could get the corresponding concepts of each entity. After this procedure, we delete some infrequent words manually, which means words that appear less than 5 records. The statistics of the corpus are summarized in Table 2.

### 4.2. Baseline and Evaluate Criterion

The objective of this paper is to verify two factor distributions impacts on overall topic distribution: the entity distribution and the background topic distribution. Since the datasets are composed of discrete records, we need to choose the baseline methods in the experiments for different purposes.

**Figure 3:**
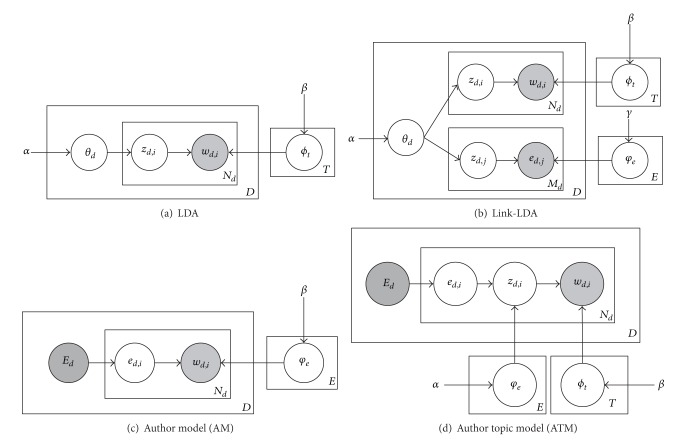
Four topic models associated with entities, topics, and words.

Firstly, we choose LDA, Link-LDA, AM, and ATM as the baselines in our experiment of testing entity distribution impact. We claim that word distributions should depend on associated entities besides the topics.  Figure 3 gives the graphical representations of these four models based on the notations of this paper.

From Figure 3, we note that besides LDA, the other three all contain entities, so they are suitable for the first test. Secondly, to verify the background topics distribution's impact, we can divide RETM into several models due to different test objectives. In other words, RETM can be simplified as LDA if we do not take entity distributions and background topic distributions into consideration. If we measure the impact caused by entities other than background topic distributions, meaning all the word distribution is decided by the local topic distributions, the model is defined as RETM-self. Because RETM is the mixture of entity distribution and topic distributions, we can assess the impacts caused by each part distinctly through all kinds of comparisons.

For* BOOK*, we set *K* = 20, *α*_0_ = *α*_1_ = 0.1, and *β* = 0.1. The fact is that one record tends to choose the local topics once this record contains attributes like* abstract, overview, review*, and so forth, which usually involves long text. Therefore, we say that one record has long text if |*N*_*d*_ | >120 and *p*_*z*_ = 0.48 through statistical calculations. For* PAPER*, we set *K* = 50, *α*_0_ = *α*_1_ = 0.1, and *β* = 0.1 and when |*N*_*d*_ | >60, we define that this record has long text; in this case, *p*_*z*_ = 0.65. Above hypermeters are applicable for LDA, Link-LDA, and AM. ATM's hypermeters are set as *α*_0_ = *α*_1_ = 50/*T* and *β* = 0.01 according to [10]'s suggestion.

In addition, we perform perplexity analysis to examine the performance of each model. Perplexity evaluates how well a probability distribution or probability model predicts a sample. The lower a trained model's perplexity is, the better the topic model is, the more flexibility one model will get. The definition of perplexity is hereby given as follows:

(14) $$\begin{array}{c} \text{Perplexity}\left(W\right)=\exp\left\{-\frac{\log P\left(D^{\text{test}}\;|\;D^{\text{train}}\right)}{\sum_{d\in D^{\text{test}}}N_{d}}\right\}, \end{array}$$

where if Φ is the hypermeters set of current model, then

(15) $$\begin{array}{c} P\left(D^{\text{test}}\;|\;D^{\text{train}}\right)=\int P\left(D^{\text{test}}\;|\;\Phi \right)P\left(\Phi \;|\;D^{\text{train}}\right)d\Phi . \end{array}$$

Formula (15) can be approximately calculated by averaging *P*(Φ | *D* ^train^)  under samples from  *P*(*D* ^test^∣Φ). Since we already known a set of entities, we can get

(16) $$\begin{array}{c} P\left(D^{\text{test}}\;|\;\Phi \right)=\underset{d\in D^{\text{test}}}{\prod }P\left(w_{d}\;|\;E_{d},\Phi \right). \end{array}$$

### 4.3. Results and Analysis

#### 4.3.1. The Impact of Entity Distribution on Topic Models

This paper claims that word distributions should depend on associated entities as well as the topics. To prove this argument, we could examine the change of word distributions over topics with a fixed entity and then over entities with a fixed topic. We choose to conduct this experiment on* PAPER,* since it does not require any information about the background topics.

Take Professor Michael I. Jordan as an example for the first case study, who is a leading researcher in machine learning and artificial intelligence. He applies Bayesian networks into machine learning and makes outstanding contributions on optimization problems in likelihood estimation.  Table 3 shows the word distributions of certain topics when picking Michael I. Jordan as the fixed entity. The first column demonstrates the top 15 words in the entity prior *φ*_*e*_ of Michael. Usually the prior distribution of entities presents one person's general methodologies or research interests. Just like the word “neural learning,” “intelligence” in the first column is conforming to Michael's research interest. In this way, the model can better describe the word distribution *φ*_*e*,*z*_ under different research topics after combining the entity prior *φ*_*e*_ and the topic prior *φ*_*z*_. In the table, the remainig three columns represent top 15 words of Michael I. Jordan's three main research topics: machine learning, neural networks, and Bayesian network. From these words, we find that the top words have varied a lot with the changes of topics even concerned about the same person. Not many words are repeated or kept in the same rank. Furthermore, Table 4 displays the word distributions with one fixed research topic (machine learning) over three related authors based on *P*(*e*∣machine learning, *ε*, *Z*). It is obvious that all three authors have published many papers on machine learning area but in different research approaches. For example, Pedro Domingo is specialized in the research of Markov logic networks whereas Judea Pearl is credited for inventing Bayesian networks and several inference methods in the models; Andrew Ng is Michael I. Jordan's student, who mainly focuses on researches about deep learning recently. Although three authors study very distinct approaches under the same research topic, which causes totally different word distributions, this difference could be measured through analysis of *P*(*w*∣*e*, *z*) based on RETM. Now we can get the conclusion that topic model that is associated with entity distribution could be more detailed and distinguishable in topic category subdivision process. In addition, it provides intuitive and understandable comparisons through *P*(*w*∣*e*, *z*), which is impossible if we are just modeling *P*(*w*∣*z*)  or  *P*(*w*∣*e*).

**Figure 4:**
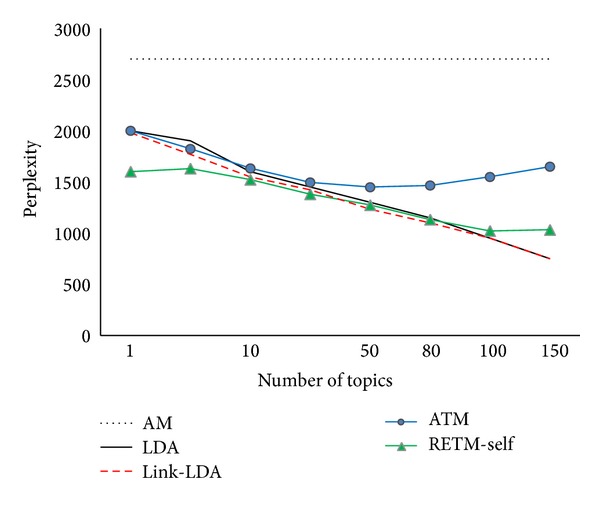
Perplexity values for different numbers of topics (*BOOK*).

We also need to analyze the perplexity value of RETM compared to the baseline models: LDA, Link-LDA, AM, and ATM. Recall that there is no background topics information in* PAPER*, so RETM and RETM-self are considered to be the same. According to (16), we randomly choose the 70% of the records as the training set *D* ^train^  and the remaining 30% records as the test set  *D* ^rest^.  Figure 4 shows the changes of perplexity of the tested models under diverse number of topics in* BOOK*. It is clear that RETM-self, LDA, and Link-LDA have very similar perplexity value, that is, mainly because most words used in academic papers are topic related, but in original corpus, not many words are associated with entity features. For instance, one author may use an inventive term that particular points to one object in his article (IoT, Cloud, etc.), this term will not be generally accepted unless such kind of research topics become popular or famous. From Figure 3(c) we can tell that AM does not have topics in its model, so it cannot gain any advantages from other priors. As a result, it gets the highest perplexity value and does not change with the increment of number of topics. Compared with other models, RETM-self has the lowest perplexity value when the number of topics is small; furthermore, the values decrease steadily as the topic numbers increase. However, it begins to rise when the topic numbers exceed 100. That is because the model has to estimate a large number of parameters, which causes overfitting problems. Same problem happens to ATM as well. Judged from the overall performances of the five models, we can get the conclusion that models that adopt the given associated entity sets as extra information to learn the topic distributions for records perform better than other models.

#### 4.3.2. The Impact of Background Topics Distribution on Topic Models

In this task, we prefer to use* BOOK* in that this dataset contains detailed linking information about records crawled from the data sources. In this case, we could easily summarize the background topic from one record as well as its neighbor records. This time, we compare RETM with LDA, ATM, and RETM-self. Since AM does not involve entity or background topics in its model, there is certainly no change in its perplexity values as the topic number grows; thus, it has no comparison value. Furthermore, LDA and Link-LDA perform very alike under these situations so we elect to select LDA as the representative model for simplicity. Other hyperparameters or experimental settings are kept the same as the previous one. Unlike* PAPER*, we need to preprocess the data in* BOOK* with DBpedia knowledge base. As a result, relatively more words that associated with entities are chosen for the corpus.

**Figure 5:**
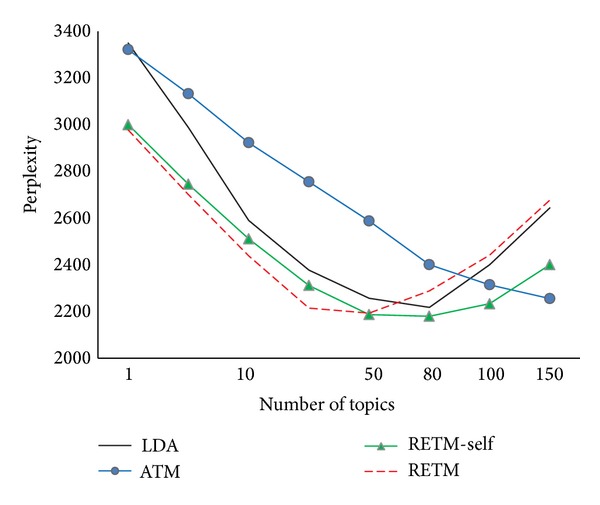
Perplexity values for different numbers of topics (*PAPER*).

Figure 5 shows the changes of perplexity of the tested models under different numbers of topics for* PAPER* dataset. First of all, ATM gives almost the same performance as in* BOOK*. It only needs to estimate a few hyperparameters and can make use of the extra entity distributions prior informatio;, its perplexity values have been decreasing as more topics added. Secondly, both RETM-self and RETM's performances are better than LDA, which further indicates that with the help of entity distributions, our model can be more accurate and convenient to categorize the given topics. This effect is rather obvious when the number of topics is no less than 20. Finally, we can find that REMT gets the lowest perplexity values, comparing RETM with RETM-self, especially when the number of topics is less than 10. No doubt RETM can take advantage of the related background topics information, which is the only difference between the two models. Unfortunately, RETM has to estimate more parameters when picking the background topics distributions, which bring a large amount of calculation and lead to enormous complexity in each sampling iteration. Consequently, its perplexity values grow very fast, limiting its generalization performance, and get worse when the number of topics is larger than 100.

Next, we will discuss to what extent the background topic distributions can affect the general topic. Recall that all the above experiments are conducted under the assumption that the record chooses to use the local topics when this record has long described text in its attributes, meanwhile, it decide to choose the background topics which generated from current record and neighbor records when the text is consist of short tokens. Now to prove the accuracy of hypothesis, we randomly pick up 2000 records that contain different lengths text from the* BOOK* as set 1,and then pick up 2000 records that do not have any* overview* information about this book manually as set 2. In this case, set 2 is considered to contain short text only. We now extract the topics of each record from two sets, respectively, and compare the accuracy with ATM and LDA. The results can be found in Table 5. We can see that for the random dataset 1, all three models perform fairly well except that LDA and ATM fail to get high points when dealing with set 2. ATM performs slightly better than LDA due to the linking information gathered from entities but still cannot compare with RETM, who gains almost twice accuracy rate than others. This further provides evidence that making use of the distributions of background topics can greatly improve the topic models, locate the correct position of each word, and finally extract the record concept that better expresses the content itself.

## 5. Conclusions

This paper studies a particular topic model construction method for the infrastructure of the information network for IoT. Based on the fact that almost every record extracted from the query result lists of IoT involves several entities, it proves that the entity distributions have some substantial impact on the word distributions within records. That is, once we add the entity distributions as priors into the topic model, the model can produce meticulous category as well as increase its distinguishability, which helps the users to understand the record content easily. In addition, our model particularly pays attention to the way each record is extracted. We classify the records gathered after querying the same keywords and then claim that the word distribution in every record should associate with topics that is generated from either its own topic distributions or the background topic distributions. These two arguments are accepted through the experiments on real-world datasets. From the analysis of experimental results, we find that our model is more suitable for extract topics from records compared to other traditional models. Furthermore, this approach can extract more accurate topics that can exactly describe the content of the records. So our work has some advanced and realistic significance to the further work of IoT.

## Figures and Tables

**Algorithm 1:**
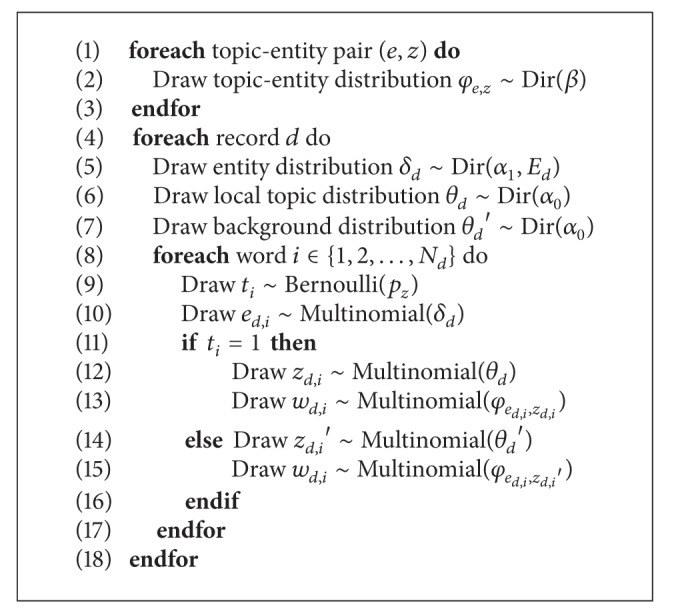
Record entity topic model (RETM).

**Table 1:** Example of query records from IMDB (movies).

Avatar	USA∣ UK	James Cameron	Twentieth Century Fox Film Corporation	January 4, 2010 (China)
Star trek	USA∣ Germany	J.J. Abrams	Paramount Pictures	May 15, 2009 (China)
Men in black 3	USA∣ United Arab Emirates	Barry Sonnenfeld	Columbia Pictures	May 25, 2012 (USA)

**Table 2:** Summary statistics of the corpus.

Dataset	*R*	*E*	*W*	avg (|*E*_*d*_|)	avg (|*N*_*d*_|)
Book	2000	622	11342	10.82	178.6
Paper	19235	3175	10889	1.76	98.61

**Table 3:** Michael I. Jordan's entity prior (*φ*_*e*_) and word distributions (*φ*_*e*,*z*_) of his research topics.

Michael I. Jordan	Machine learning	Neural networks	Bayesian network
Learning	Probabilistic	Cognitive	Causal
Neural	Causal	Learn	Distributions
Processing	Systems	Neural	Models
Awards	Methods	Networks	Markovian
Advances	Reasoning	Proceedings	dynamic
Machine	Algorithm	Artificial	Identification
AAAI	Graphs	Conference	Characterization
Bayesian	Model	International	Recursive
ACM	Kernel	System	Joint
Networks	Computational	NIPS	Variables
Statistical	Award	Graph	Data
Computational	Uncertainty	Control	Effects
Associates	Representation	Kernel	Algorithm
Conference	Processing	Science	Based
Intelligence	Networks	Probability	Clustering

**Table 4:** Machine learning's topic prior (*φ*_*z*_) and word distributions (*φ*_*e*,*z*_) of its related entities.

Machine learning	Judea Pearl	Pedro Domingos	Andrew Ng
Learning	Causal	Logic	Learning
Machine	Revisited	Markov	Stanford
Data	Markovian	Networks	Neural
Algorithm	Data	Learning	Deep
Representation	Counterfactual	MLNs	Networks
Theory	Artificial	Algorithm	Word
Examples	Explanations	Theory	Technology
Sparse	Independence	Knowledge	Model
Analysis	Path	Order	Google
Computational	Specificity	Models	Class
Artificial	Representation	Systems	Machine
Recognition	Proven	Representation	Artificial
Programming	Tags	Reasoning	Intelligence
Model	Embracing	World	System
Supervised	Tolerating	Structure	Data

**Table 5:** Accuracy rate of topics models for records with different lengths.

Name	Manual	Random
ATM	0.341	0.72
LDA	0.311	0.695
RETM	**0.822**	0.738

## Notations

*R*: Number of query records
*K*: Number of topic-entity pairs
*W*: Number of words
*E*: Number of entities
*N*_*d*_: Number of word tokens in record *d*, *d* ∈ {1,2,…, *R*}
*K*_*c*_: Number of topics produced by current record
*K*_*b*_: Number of topics produced by neighbor records
*θ*_*d*_: Multinomial distribution of topics specific to record *d*
*θ*_*d*_′: Multinomial distribution of overall topics specific to record *d*
*δ*_*d*_: Multinomial distribution of entities specific to record *d*
*E*_*d*_: List of entities associated with record *d*
*e*_*d*,*i*_: Entity associated with the *i*th token in record *d*
*z*_*d*,*i*_: Topic associated with the *i*th token in record *d*
*w*_*d*,*i*_: *i*th token in record *d*
*φ*_*e*,*z*_: Multinomial distribution of words specific to entity *e* and topic *t*
*P*_*z*_: Probability of the final topics coming from record *d* itself
*t*: Flag variables
*Z*: Set of all topic assignments {*z*_*d*,*i*_}
*ε*: Set of all entities assignments {*e*_*d*,*i*_}
Φ: Set of all parameters in the model like *α*_0_, *α*_1_, *β*.
